# [μ-14,29-Di-*tert*-butyl-3,10,18,25-tetra­azatpenta­cyclo­[25.3.1.1^12,16^.0^4,9^.0^19,24^]dotriaconta-1(31),4,6,8,12(32),14,16,19,21,23,27,29-dodeca­ene-31,32-diol­ato]bis­[(nitrato-κ^2^ *O*,*O*′)zinc(II)]

**DOI:** 10.1107/S1600536809028530

**Published:** 2009-07-25

**Authors:** Li-Jing Fan, Jian-Fang Ma, Bo Liu

**Affiliations:** a Department of Chemistry, Northeast Normal University, Changchun 130024, People’s Republic of China

## Abstract

In the title centrosymmetric dinuclear zinc(II) complex, [Zn_2_(C_36_H_42_N_4_O_2_)(NO_3_)_2_], the Zn^II^ atom has a distorted octa­hedral geometry, defined by two N atoms and two O atoms from the macrocyclic ligand and two O atoms from a chelating nitrate anion and are bridged by two phenolate O atoms, forming a four-membered Zn_2_O_2_ ring.

## Related literature

For general background to the biochemistry of zinc(II) compounds, see: Bazzicalupi *et al.* (1997 ▶); Burley *et al.* (1990 ▶); Lipscomb & Straeter (1996 ▶); Roderick & Mathews (1993 ▶). For related structures, see: Dutta *et al.* (2005 ▶). For further synthetic details, see: Fan *et al.* (2009 ▶).
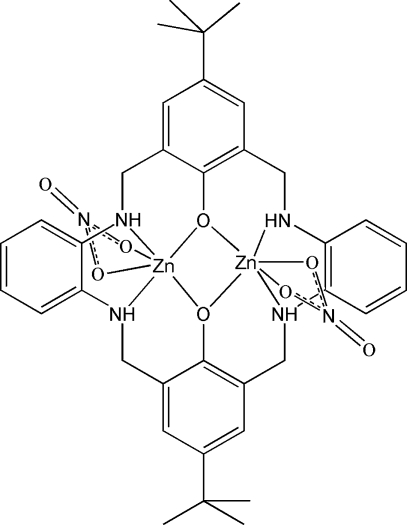


## Experimental

### 

#### Crystal data

[Zn_2_(C_36_H_42_N_4_O_2_)(NO_3_)_2_]
*M* *_r_* = 817.20
Monoclinic, 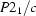

*a* = 13.7149 (8) Å
*b* = 18.0691 (10) Å
*c* = 7.3523 (3) Å
β = 101.110 (5)°
*V* = 1787.87 (16) Å^3^
*Z* = 2
Mo *K*α radiation
μ = 1.40 mm^−1^
*T* = 293 K
0.45 × 0.25 × 0.20 mm

#### Data collection

Oxford Diffraction Gemini R Ultra diffractometer
Absorption correction: multi-scan (*CrysAlis RED*; Oxford Diffraction, 2006 ▶) *T*_min_ = 0.661, *T*_max_ = 0.752
15034 measured reflections
4340 independent reflections
1698 reflections with *I* > 2σ(*I*)
*R*_int_ = 0.099

#### Refinement

*R*[*F* ^2^ > 2σ(*F* ^2^)] = 0.049
*wR*(*F* ^2^) = 0.065
*S* = 0.91
4340 reflections
241 parameters
357 restraints
H atoms treated by a mixture of independent and constrained refinement
Δρ_max_ = 0.66 e Å^−3^
Δρ_min_ = −0.45 e Å^−3^

### 

Data collection: *CrysAlis CCD* (Oxford Diffraction, 2006 ▶); cell refinement: *CrysAlis RED* (Oxford Diffraction, 2006 ▶); data reduction: *CrysAlis RED*; program(s) used to solve structure: *SHELXS97* (Sheldrick, 2008 ▶); program(s) used to refine structure: *SHELXL97* (Sheldrick, 2008 ▶); molecular graphics: *SHELXTL* (Sheldrick, 2008 ▶); software used to prepare material for publication: *SHELXTL*.

## Supplementary Material

Crystal structure: contains datablocks I, global. DOI: 10.1107/S1600536809028530/hy2213sup1.cif

Structure factors: contains datablocks I. DOI: 10.1107/S1600536809028530/hy2213Isup2.hkl

Additional supplementary materials:  crystallographic information; 3D view; checkCIF report

## Figures and Tables

**Table 1:** Selected bond lengths (Å)

Zn1—N1	2.081 (4)
Zn1—N2	2.102 (3)
Zn1—O1	2.264 (3)
Zn1—O2	2.243 (3)
Zn1—O4	2.019 (2)
Zn1—O4^i^	2.043 (2)

Symmetry code: (i) 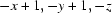.

**Fig. 1.**
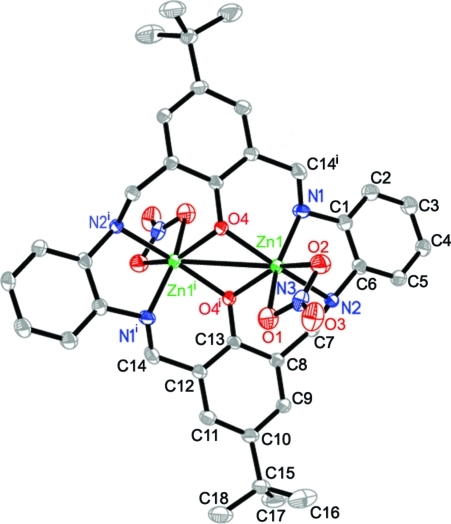
Molecular structure of the title compound. Displaceement ellipsoids are draw at the 30% probability level. H atoms have been omitted for clarity.

## supplementary crystallographic information

### Comment

Dinuclear zinc(II) compounds have attracted much interest as a result of their significance in biological systems (Burley *et al.*, 1990; Roderick & Mathews, 1993). In addition, some synthetic dinuclear zinc(II) compounds are found to have functions in dephosphorylation (Bazzicalupi *et al.*, 1997). As part of our studies in this area, the title compound, a new dinuclear zinc(II) compound, has been synthesized and its structure is reported here (Fig. 1).

In the title centrosymmetric dinuclear zinc(II) compound, each of the two Zn^II^ atoms has a distorted octahedral geometry, defined by two N atoms and two O atoms from the macrocyclic (C_36_H_42_N_4_O_2_) ligand and two O atoms from a chelating nitrate anion. The two Zn atoms are bridged by two phenolate O atoms, forming a four-membered Zn_2_O_2_ ring. The Zn—O and Zn—N distances are normal (Table 1) (Dutta *et al.*, 2005).

### Experimental

The title compound was prepared by a reaction between the macrocyclic ligand C_36_H_44_N_4_O_2_ (H_2_L), which was synthesized according to the published procedure (Fan *et al.*, 2009), and zinc nitrate. A mixture of H_2_L (0.135 g, 0.25 mmol) and Zn(NO_3_)_2_.6H_2_O (0.149 g, 0.5 mmol) in ethanol (20 ml) was heated with stirring to yield a clear pale yellow solution. Filtration and cooling to room temperature resulted in the formation of a crystalline precipitate. Recrystallization by slow evaporation of an ethanol solution of the compound resulted in well formed yellow blocks of the title compound (yield 52%).

### Refinement

N-bonded H atoms were located in a difference map and their coordinates were freely refined, with *U*_iso_ fixed. C-bonded H atoms were positioned geometrically and refined as riding atoms, with C—H = 0.93–0.96 Å and with *U*_iso_(H) = 1.2(or 1.5 for methyl)*U*_eq_(C).

### Crystal data

**Table:**

[Zn_2_(C_36_H_42_N_4_O_2_)(NO_3_)_2_]	*F*(000) = 848
*M**_r_* = 817.20	*D*_x_ = 1.518 Mg m^−^^3^
Monoclinic, *P*2_1_/*c*	Mo *K*α radiation, λ = 0.71073 Å
Hall symbol: -P 2ybc	Cell parameters from 2682 reflections
*a* = 13.7149 (8) Å	θ = 1.9–29.2°
*b* = 18.0691 (10) Å	µ = 1.40 mm^−^^1^
*c* = 7.3523 (3) Å	*T* = 293 K
β = 101.110 (5)°	Block, yellow
*V* = 1787.87 (16) Å^3^	0.45 × 0.25 × 0.20 mm
*Z* = 2	

### Data collection

**Table:**

Oxford Diffraction Gemini R Ultra diffractometer	4340 independent reflections
Radiation source: fine-focus sealed tube	1698 reflections with *I* > 2σ(*I*)
graphite	*R*_int_ = 0.099
Detector resolution: 10.0 pixels mm^-1^	θ_max_ = 29.3°, θ_min_ = 1.9°
ω scans	*h* = −16→17
Absorption correction: multi-scan (*CrysAlis RED*; Oxford Diffraction, 2006)	*k* = −24→24
*T*_min_ = 0.661, *T*_max_ = 0.752	*l* = −8→10
15034 measured reflections	

### Refinement

**Table:**

Refinement on *F*^2^	Primary atom site location: structure-invariant direct methods
Least-squares matrix: full	Secondary atom site location: difference Fourier map
*R*[*F*^2^ > 2σ(*F*^2^)] = 0.049	Hydrogen site location: inferred from neighbouring sites
*wR*(*F*^2^) = 0.065	H atoms treated by a mixture of independent and constrained refinement
*S* = 0.91	*w* = 1/[σ^2^(*F*_o_^2^) + (0.01*P*)^2^] where *P* = (*F*_o_^2^ + 2*F*_c_^2^)/3
4340 reflections	(Δ/σ)_max_ = 0.001
241 parameters	Δρ_max_ = 0.66 e Å^−^^3^
357 restraints	Δρ_min_ = −0.45 e Å^−^^3^

### Fractional atomic coordinates and isotropic or equivalent isotropic displacement parameters (Å^2^)

**Table:**

	*x*	*y*	*z*	*U*_iso_*/*U*_eq_	
C1	0.2727 (3)	0.6078 (2)	0.1435 (5)	0.0401 (11)	
C2	0.1724 (3)	0.6150 (2)	0.1476 (5)	0.0486 (12)	
H2	0.1254	0.5897	0.0621	0.058*	
C3	0.1424 (3)	0.6598 (2)	0.2783 (5)	0.0506 (12)	
H3	0.0753	0.6651	0.2807	0.061*	
C4	0.2122 (4)	0.6965 (2)	0.4047 (5)	0.0469 (12)	
H4	0.1920	0.7266	0.4931	0.056*	
C5	0.3108 (3)	0.6893 (2)	0.4022 (5)	0.0404 (11)	
H5	0.3572	0.7141	0.4900	0.048*	
C6	0.3426 (3)	0.6460 (2)	0.2723 (5)	0.0332 (10)	
C7	0.5006 (3)	0.4041 (2)	−0.4386 (4)	0.0334 (11)	
H7A	0.4997	0.3731	−0.5467	0.040*	
H7B	0.5382	0.4484	−0.4537	0.040*	
C8	0.3954 (3)	0.4258 (2)	−0.4294 (4)	0.0296 (9)	
C9	0.3152 (3)	0.3961 (2)	−0.5486 (5)	0.0386 (10)	
H9	0.3268	0.3600	−0.6320	0.046*	
C10	0.2177 (3)	0.4175 (3)	−0.5504 (5)	0.0427 (10)	
C11	0.2043 (3)	0.4716 (2)	−0.4249 (5)	0.0464 (11)	
H11	0.1400	0.4879	−0.4243	0.056*	
C12	0.2823 (3)	0.5029 (2)	−0.2995 (5)	0.0375 (11)	
C13	0.3800 (3)	0.4796 (2)	−0.3002 (5)	0.0322 (10)	
C14	0.2598 (3)	0.5610 (3)	−0.1703 (5)	0.0584 (12)	
H14A	0.2707	0.6085	−0.2244	0.070*	
H14B	0.1894	0.5578	−0.1684	0.070*	
C15	0.1319 (3)	0.3808 (3)	−0.6848 (6)	0.0525 (12)	
C16	0.1355 (4)	0.2980 (3)	−0.6507 (6)	0.0896 (16)	
H16A	0.1987	0.2791	−0.6668	0.134*	
H16B	0.0835	0.2744	−0.7372	0.134*	
H16C	0.1267	0.2882	−0.5266	0.134*	
C17	0.1414 (3)	0.3929 (2)	−0.8848 (5)	0.0749 (14)	
H17A	0.2044	0.3745	−0.9031	0.112*	
H17B	0.1367	0.4448	−0.9128	0.112*	
H17C	0.0889	0.3670	−0.9653	0.112*	
C18	0.0325 (3)	0.4077 (3)	−0.6600 (6)	0.0918 (16)	
H18A	0.0253	0.3993	−0.5344	0.138*	
H18B	−0.0185	0.3813	−0.7427	0.138*	
H18C	0.0268	0.4596	−0.6869	0.138*	
N1	0.3096 (3)	0.5622 (2)	0.0108 (5)	0.0479 (11)	
N2	0.4476 (3)	0.63682 (17)	0.2695 (4)	0.0315 (9)	
N3	0.5574 (4)	0.6847 (2)	−0.1030 (5)	0.0470 (12)	
O1	0.6025 (2)	0.63223 (18)	−0.0177 (4)	0.0545 (8)	
O2	0.4639 (3)	0.68288 (18)	−0.1261 (4)	0.0575 (10)	
O3	0.5992 (3)	0.73463 (18)	−0.1672 (4)	0.0714 (11)	
O4	0.4592 (2)	0.50597 (14)	−0.1821 (3)	0.0309 (7)	
Zn1	0.46177 (4)	0.57671 (3)	0.03129 (6)	0.03462 (15)	
H1N	0.296 (3)	0.5176 (13)	0.031 (5)	0.052*	
H2N	0.475 (3)	0.6800 (13)	0.282 (5)	0.052*	

### Atomic displacement parameters (Å^2^)

**Table:**

	*U*^11^	*U*^22^	*U*^33^	*U*^12^	*U*^13^	*U*^23^
C1	0.034 (3)	0.050 (3)	0.040 (2)	0.006 (2)	0.017 (2)	−0.011 (2)
C2	0.037 (3)	0.065 (3)	0.045 (2)	0.003 (2)	0.012 (2)	−0.008 (2)
C3	0.038 (3)	0.063 (3)	0.055 (3)	0.006 (2)	0.019 (2)	−0.010 (2)
C4	0.046 (3)	0.052 (3)	0.045 (2)	0.011 (3)	0.014 (2)	−0.014 (2)
C5	0.038 (3)	0.045 (3)	0.039 (2)	0.003 (2)	0.010 (2)	−0.010 (2)
C6	0.029 (3)	0.041 (3)	0.032 (2)	0.009 (2)	0.012 (2)	0.0007 (19)
C7	0.036 (3)	0.039 (3)	0.026 (2)	0.004 (2)	0.0078 (19)	−0.0020 (19)
C8	0.030 (2)	0.033 (2)	0.0254 (19)	−0.003 (2)	0.0060 (18)	−0.003 (2)
C9	0.041 (2)	0.044 (2)	0.0300 (19)	0.001 (2)	0.0052 (19)	−0.0112 (18)
C10	0.033 (2)	0.052 (2)	0.041 (2)	−0.005 (2)	0.0022 (18)	−0.013 (2)
C11	0.031 (2)	0.063 (3)	0.043 (2)	0.003 (2)	0.004 (2)	−0.010 (2)
C12	0.035 (2)	0.042 (2)	0.034 (2)	0.004 (2)	0.003 (2)	−0.0104 (19)
C13	0.031 (2)	0.038 (2)	0.028 (2)	0.001 (2)	0.007 (2)	−0.0019 (19)
C14	0.043 (3)	0.078 (3)	0.051 (2)	0.020 (2)	0.001 (2)	−0.018 (2)
C15	0.034 (3)	0.061 (3)	0.061 (2)	0.001 (2)	0.006 (2)	−0.019 (2)
C16	0.083 (3)	0.087 (3)	0.088 (3)	−0.027 (3)	−0.011 (3)	−0.007 (3)
C17	0.064 (3)	0.089 (3)	0.062 (3)	−0.011 (3)	−0.013 (2)	−0.013 (3)
C18	0.048 (3)	0.120 (4)	0.101 (3)	−0.012 (3)	−0.002 (3)	−0.055 (3)
N1	0.032 (2)	0.065 (3)	0.046 (2)	0.000 (3)	0.0058 (19)	−0.017 (3)
N2	0.037 (3)	0.028 (2)	0.0292 (18)	0.004 (2)	0.0064 (18)	−0.0054 (17)
N3	0.071 (4)	0.031 (3)	0.041 (2)	0.005 (3)	0.018 (3)	−0.005 (2)
O1	0.058 (2)	0.046 (2)	0.062 (2)	−0.0080 (17)	0.0188 (18)	0.0047 (17)
O2	0.055 (3)	0.063 (3)	0.0534 (19)	0.003 (2)	0.007 (2)	−0.0025 (17)
O3	0.098 (3)	0.038 (2)	0.090 (2)	−0.012 (2)	0.046 (2)	0.0090 (19)
O4	0.032 (2)	0.0326 (18)	0.0283 (15)	0.0044 (15)	0.0067 (14)	−0.0065 (13)
Zn1	0.0343 (3)	0.0372 (3)	0.0319 (2)	0.0038 (4)	0.0052 (2)	−0.0047 (3)

### Geometric parameters (Å, °)

**Table:**

C1—C2	1.388 (5)	C14—H14A	0.9700
C1—C6	1.393 (5)	C14—H14B	0.9700
C1—N1	1.441 (5)	C15—C18	1.490 (5)
C2—C3	1.378 (5)	C15—C16	1.516 (6)
C2—H2	0.9300	C15—C17	1.517 (5)
C3—C4	1.369 (5)	C16—H16A	0.9600
C3—H3	0.9300	C16—H16B	0.9600
C4—C5	1.363 (5)	C16—H16C	0.9600
C4—H4	0.9300	C17—H17A	0.9600
C5—C6	1.368 (5)	C17—H17B	0.9600
C5—H5	0.9300	C17—H17C	0.9600
C6—N2	1.453 (5)	C18—H18A	0.9600
C7—N2^i^	1.502 (4)	C18—H18B	0.9600
C7—C8	1.510 (5)	C18—H18C	0.9600
C7—H7A	0.9700	Zn1—N1	2.081 (4)
C7—H7B	0.9700	N1—H1N	0.845 (19)
C8—C9	1.376 (5)	N2—C7^i^	1.502 (4)
C8—C13	1.404 (5)	Zn1—N2	2.102 (3)
C9—C10	1.390 (5)	N2—H2N	0.863 (19)
C9—H9	0.9300	N3—O3	1.212 (4)
C10—C11	1.380 (5)	N3—O1	1.234 (4)
C10—C15	1.534 (5)	N3—O2	1.262 (5)
C11—C12	1.391 (5)	Zn1—O1	2.264 (3)
C11—H11	0.9300	Zn1—O2	2.243 (3)
C12—C13	1.405 (5)	Zn1—O4	2.019 (2)
C12—C14	1.487 (5)	Zn1—O4^i^	2.043 (2)
C13—O4	1.341 (4)	Zn1—Zn1^i^	3.0302 (10)
C14—N1	1.375 (4)		
			
C2—C1—C6	119.6 (4)	C15—C16—H16C	109.5
C2—C1—N1	123.1 (4)	H16A—C16—H16C	109.5
C6—C1—N1	117.3 (4)	H16B—C16—H16C	109.5
C3—C2—C1	120.0 (4)	C15—C17—H17A	109.5
C3—C2—H2	120.0	C15—C17—H17B	109.5
C1—C2—H2	120.0	H17A—C17—H17B	109.5
C4—C3—C2	119.6 (4)	C15—C17—H17C	109.5
C4—C3—H3	120.2	H17A—C17—H17C	109.5
C2—C3—H3	120.2	H17B—C17—H17C	109.5
C3—C4—C5	120.7 (4)	C15—C18—H18A	109.5
C3—C4—H4	119.7	C15—C18—H18B	109.5
C5—C4—H4	119.7	H18A—C18—H18B	109.5
C4—C5—C6	120.9 (4)	C15—C18—H18C	109.5
C4—C5—H5	119.5	H18A—C18—H18C	109.5
C6—C5—H5	119.5	H18B—C18—H18C	109.5
C5—C6—C1	119.2 (4)	C14—N1—C1	119.5 (4)
C5—C6—N2	121.6 (4)	C14—N1—Zn1	112.1 (3)
C1—C6—N2	119.1 (4)	C1—N1—Zn1	110.9 (3)
N2^i^—C7—C8	113.3 (3)	C14—N1—H1N	94 (3)
N2^i^—C7—H7A	108.9	C1—N1—H1N	108 (3)
C8—C7—H7A	108.9	Zn1—N1—H1N	111 (3)
N2^i^—C7—H7B	108.9	C6—N2—C7^i^	110.9 (3)
C8—C7—H7B	108.9	C6—N2—Zn1	108.8 (2)
H7A—C7—H7B	107.7	C7^i^—N2—Zn1	109.3 (2)
C9—C8—C13	119.6 (4)	C6—N2—H2N	108 (3)
C9—C8—C7	121.6 (4)	C7^i^—N2—H2N	103 (3)
C13—C8—C7	118.7 (4)	Zn1—N2—H2N	116 (3)
C8—C9—C10	123.2 (4)	O3—N3—O1	122.8 (5)
C8—C9—H9	118.4	O3—N3—O2	120.7 (5)
C10—C9—H9	118.4	O1—N3—O2	116.4 (5)
C11—C10—C9	116.3 (4)	N3—O1—Zn1	93.6 (3)
C11—C10—C15	123.5 (4)	N3—O2—Zn1	93.8 (3)
C9—C10—C15	120.2 (4)	C13—O4—Zn1	128.2 (2)
C10—C11—C12	123.1 (4)	C13—O4—Zn1^i^	111.8 (2)
C10—C11—H11	118.5	Zn1—O4—Zn1^i^	96.50 (9)
C12—C11—H11	118.5	O4—Zn1—O4^i^	83.50 (9)
C11—C12—C13	119.2 (4)	O4—Zn1—N1	89.82 (13)
C11—C12—C14	118.9 (4)	O4^i^—Zn1—N1	111.54 (13)
C13—C12—C14	121.9 (4)	O4—Zn1—N2	169.86 (13)
O4—C13—C12	123.1 (4)	O4^i^—Zn1—N2	92.88 (11)
O4—C13—C8	118.4 (4)	N1—Zn1—N2	82.68 (14)
C12—C13—C8	118.6 (4)	O4—Zn1—O2	98.08 (11)
N1—C14—C12	120.3 (4)	O4^i^—Zn1—O2	147.90 (13)
N1—C14—H14A	107.2	N1—Zn1—O2	100.54 (14)
C12—C14—H14A	107.2	N2—Zn1—O2	90.02 (12)
N1—C14—H14B	107.2	O4—Zn1—O1	92.56 (11)
C12—C14—H14B	107.2	O4^i^—Zn1—O1	91.78 (11)
H14A—C14—H14B	106.9	N1—Zn1—O1	156.68 (13)
C18—C15—C16	107.6 (4)	N2—Zn1—O1	97.03 (12)
C18—C15—C17	108.8 (4)	O2—Zn1—O1	56.16 (11)
C16—C15—C17	107.2 (4)	O4—Zn1—Zn1^i^	42.06 (7)
C18—C15—C10	112.8 (4)	O4^i^—Zn1—Zn1^i^	41.45 (6)
C16—C15—C10	108.9 (4)	N1—Zn1—Zn1^i^	104.20 (11)
C17—C15—C10	111.4 (4)	N2—Zn1—Zn1^i^	133.61 (9)
C15—C16—H16A	109.5	O2—Zn1—Zn1^i^	131.67 (9)
C15—C16—H16B	109.5	O1—Zn1—Zn1^i^	92.91 (9)
H16A—C16—H16B	109.5		
			
C6—C1—C2—C3	0.1 (6)	C12—C13—O4—Zn1	4.7 (5)
N1—C1—C2—C3	−179.9 (4)	C8—C13—O4—Zn1	−174.7 (2)
C1—C2—C3—C4	−0.6 (6)	C12—C13—O4—Zn1^i^	122.7 (4)
C2—C3—C4—C5	0.2 (7)	C8—C13—O4—Zn1^i^	−56.6 (4)
C3—C4—C5—C6	0.8 (7)	C13—O4—Zn1—O4^i^	124.4 (3)
C4—C5—C6—C1	−1.2 (6)	Zn1^i^—O4—Zn1—O4^i^	0.0
C4—C5—C6—N2	−179.0 (4)	C13—O4—Zn1—N1	12.7 (3)
C2—C1—C6—C5	0.8 (6)	Zn1^i^—O4—Zn1—N1	−111.71 (13)
N1—C1—C6—C5	−179.2 (4)	C13—O4—Zn1—N2	54.8 (8)
C2—C1—C6—N2	178.7 (4)	Zn1^i^—O4—Zn1—N2	−69.6 (7)
N1—C1—C6—N2	−1.4 (6)	C13—O4—Zn1—O2	−87.9 (3)
N2^i^—C7—C8—C9	−114.2 (4)	Zn1^i^—O4—Zn1—O2	147.67 (13)
N2^i^—C7—C8—C13	68.2 (5)	C13—O4—Zn1—O1	−144.1 (3)
C13—C8—C9—C10	0.9 (6)	Zn1^i^—O4—Zn1—O1	91.50 (12)
C7—C8—C9—C10	−176.7 (4)	C13—O4—Zn1—Zn1^i^	124.4 (3)
C8—C9—C10—C11	0.4 (6)	C14—N1—Zn1—O4	−41.6 (3)
C8—C9—C10—C15	−178.8 (4)	C1—N1—Zn1—O4	−177.9 (3)
C9—C10—C11—C12	−1.3 (6)	C14—N1—Zn1—O4^i^	−124.5 (3)
C15—C10—C11—C12	177.8 (4)	C1—N1—Zn1—O4^i^	99.2 (3)
C10—C11—C12—C13	0.9 (6)	C14—N1—Zn1—N2	145.2 (3)
C10—C11—C12—C14	179.8 (4)	C1—N1—Zn1—N2	8.9 (3)
C11—C12—C13—O4	−178.9 (3)	C14—N1—Zn1—O2	56.6 (3)
C14—C12—C13—O4	2.2 (6)	C1—N1—Zn1—O2	−79.7 (3)
C11—C12—C13—C8	0.5 (6)	C14—N1—Zn1—O1	54.5 (5)
C14—C12—C13—C8	−178.4 (4)	C1—N1—Zn1—O1	−81.8 (4)
C9—C8—C13—O4	178.1 (3)	C14—N1—Zn1—Zn1^i^	−81.5 (3)
C7—C8—C13—O4	−4.3 (5)	C1—N1—Zn1—Zn1^i^	142.2 (2)
C9—C8—C13—C12	−1.3 (6)	C6—N2—Zn1—O4	−51.9 (8)
C7—C8—C13—C12	176.3 (3)	C7^i^—N2—Zn1—O4	69.3 (8)
C11—C12—C14—N1	140.1 (4)	C6—N2—Zn1—O4^i^	−120.7 (2)
C13—C12—C14—N1	−40.9 (7)	C7^i^—N2—Zn1—O4^i^	0.5 (2)
C11—C10—C15—C18	−2.3 (6)	C6—N2—Zn1—N1	−9.4 (2)
C9—C10—C15—C18	176.8 (4)	C7^i^—N2—Zn1—N1	111.8 (3)
C11—C10—C15—C16	−121.6 (5)	C6—N2—Zn1—O2	91.3 (3)
C9—C10—C15—C16	57.5 (5)	C7^i^—N2—Zn1—O2	−147.6 (2)
C11—C10—C15—C17	120.4 (4)	C6—N2—Zn1—O1	147.1 (2)
C9—C10—C15—C17	−60.5 (6)	C7^i^—N2—Zn1—O1	−91.7 (2)
C12—C14—N1—C1	−166.4 (4)	C6—N2—Zn1—Zn1^i^	−112.0 (2)
C12—C14—N1—Zn1	61.4 (5)	C7^i^—N2—Zn1—Zn1^i^	9.2 (3)
C2—C1—N1—C14	40.4 (6)	N3—O2—Zn1—O4	−88.6 (2)
C6—C1—N1—C14	−139.5 (4)	N3—O2—Zn1—O4^i^	2.0 (3)
C2—C1—N1—Zn1	173.0 (3)	N3—O2—Zn1—N1	−180.0 (2)
C6—C1—N1—Zn1	−6.9 (5)	N3—O2—Zn1—N2	97.5 (2)
C5—C6—N2—C7^i^	66.3 (5)	N3—O2—Zn1—O1	−1.0 (2)
C1—C6—N2—C7^i^	−111.5 (4)	N3—O2—Zn1—Zn1^i^	−60.0 (3)
C5—C6—N2—Zn1	−173.5 (3)	N3—O1—Zn1—O4	99.0 (2)
C1—C6—N2—Zn1	8.7 (4)	N3—O1—Zn1—O4^i^	−177.4 (2)
O3—N3—O1—Zn1	−179.5 (4)	N3—O1—Zn1—N1	3.5 (5)
O2—N3—O1—Zn1	−1.7 (4)	N3—O1—Zn1—N2	−84.3 (2)
O3—N3—O2—Zn1	179.5 (3)	N3—O1—Zn1—O2	1.0 (2)
O1—N3—O2—Zn1	1.7 (4)	N3—O1—Zn1—Zn1^i^	141.1 (2)

Symmetry codes: (i) −*x*+1, −*y*+1, −*z*.
